# Sex-Independent Upregulation of miR-146a-5p in Parkinson’s Disease Patients: A Longitudinal Study

**DOI:** 10.3390/ijms262110315

**Published:** 2025-10-23

**Authors:** Annamaria Vallelunga, Tommaso Iannitti, Giovanna Dati, Julio César Morales-Medina, Marina Picillo, Marianna Amboni, Calogero Edoardo Cicero, Roberto Cilia, Rosa De Micco, Anna De Rosa, Alessio Di Fonzo, Roberto Eleopra, Augusta Giglio, Giulia Lazzeri, Alessandra Nicoletti, Claudio Pacchetti, Andrea Soricelli, Alessandro Tessitore, Roberta Zangaglia, Paolo Barone, Maria Teresa Pellecchia

**Affiliations:** 1 Centro de Investigación en Reproducción Animal, CINVESTAV-Universidad Autónoma de Tlaxcala, AP 62, Tlaxcala CP 90000, Mexico; 2 Section of Experimental Medicine, Department of Medical Sciences, University of Ferrara, Via Fossato di Mortara 70, 44121 Ferrara, Italy; 3 Department of Medicine, Surgery and Dentistry “Scuola Medica Salernitana”, Neuroscience Section, University of Salerno, 84131 Salerno, Italy; giovannadati@hotmail.it (G.D.); mamboni@unisa.it (M.A.); pbarone@unisa.it (P.B.); mpellecchia@unisa.it (M.T.P.); 4 Laboratorio de Neuropsiquiatria, Instituto de Fisiología, Benemérita Universidad Autónoma de Puebla, Puebla 72570, Mexico; 5 Neurologic Unit, AOU “Policlinico-San Marco”, Department of Medical, Surgical Sciences and Advanced Technologies, GF Ingrassia, University of Catania, 95125 Catania, Italy; edoardo.cicero@gmail.com (C.E.C.); alessandra.nicoletti@unict.it (A.N.); 6 Parkinson and Movement Disorders Unit, Department of Clinical Neurosciences, Fondazione IRCCS Istituto Neurologico Carlo Besta, 20133 Milano, Italy; roberto.cilia@istituto-besta.it (R.C.); roberto.eleopra@istituto-besta.it (R.E.); 7 Department of Advanced Medical and Surgical Sciences, University of Campania Luigi Vanvitelli, 80138 Napoli, Italy; rosita.demicco@gmail.com (R.D.M.); alessandro.tessitore@unicampania.it (A.T.); 8 Department of Neurosciences and Reproductive and Odontostomatological Sciences, Federico II University, 80131 Naples, Italy; anna.derosa1@unina.it (A.D.R.); augustagiglio@gmail.com (A.G.); 9 Neurology Unit, IRCCS Ca’ Granda Ospedale Maggiore Policlinico, 20122 Milan, Italy; alessio.difonzo@policlinico.mi.it; 10 Centro Parkinson e Parkinsonismi, ASST G. Pini-CTO, 20122 Milano, Italy; giulia.lazzeri@outlook.com; 11 Parkinson’s Disease and Movement Disorders Unit, IRCCS Mondino Foundation, 27100 Pavia, Italy; claudio.pacchetti@mondino.it (C.P.); roberta.zangaglia@mondino.it (R.Z.); 12 IRCCS SYNLAB SDN, 80143 Naples, Italy; andrea.soricelli@uniparthenope.it

**Keywords:** sex and gender, personalized medicine, sex specific evidence, sex specific differences, Parkinson’s disease, miRNAs, biomarkers

## Abstract

Parkinson’s disease (PD) is a progressive neurodegenerative disorder characterized by motor and non-motor symptoms. The absence of reliable fluid biomarkers continues to hinder early diagnosis and effective monitoring of disease progression. Circulating microRNAs (cmiRNAs) are potential candidates, given their stability in biofluids and their ability to mirror pathological processes. We conducted a longitudinal study in 30 early-stage levodopa-naive PD patients (22 men, 8 women). Serum samples were collected at baseline (T0) and at a follow-up time point two years later (T2). A panel of MicroRNAs (miRNAs) (miR-146a-5p, miR-34a-5p, miR-155-5p, miR-29a-3p, miR-106a-5p) were quantified by quantitative real-time PCR. Data were expressed as relative expression (2^−ΔCt), and statistical analyses included sex-stratified comparisons and paired tests for longitudinal changes. At baseline, no significant differences were found in the expression of the miRNAs between male and female PD patients. In contrast, longitudinal within-subject analysis revealed a highly significant upregulation in miR-146a-5p expression from T0 to T2 in both sexes (*p* < 0.0001). No other miRNAs in the panel exhibited significant changes over time. CmiR-146a-5p levels rise markedly over time in PD patients, independent of sex, suggesting that this miRNA could be a dynamic biomarker of disease progression.

## 1. Introduction

Parkinson’s disease (PD) is a progressive neurodegenerative disorder characterized by motor symptoms (bradykinesia, tremor, rigidity) and a range of non-motor features. Pathologically, PD involves the loss of dopaminergic neurons in the substantia nigra and widespread aggregation of α-synuclein, along with chronic neuroinflammation and other pathogenic processes [1]. Despite advances in understanding PD pathogenesis, there are still no reliable fluid biomarkers for diagnosing PD or tracking its progression, and clinicians rely mainly on clinical examinations. Identifying accessible biomarkers is a priority, particularly to monitor disease progression and to differentiate PD from related syndromes [2].

In recent years, small non-coding RNAs known as microRNAs (miRNAs) have emerged as promising biomarker candidates in neurodegenerative diseases. MiRNAs regulate gene expression post-transcriptionally and can reflect ongoing pathophysiological processes [3]. They are present in circulating biofluids and are remarkably stable and easy to measure in blood samples. Numerous studies have documented dysregulated miRNA profiles in PD patients compared to healthy controls [4]. For example, inflammation-associated miR-155 is elevated in blood of PD patients, whereas miR-146a-5p has been reported to be reduced compared to controls, highlighting how PD involves an imbalance of immune-related miRNA regulation [5]. Many circulating miRNAs (cmiRNAs) have been linked to PD-related pathways (e.g., oxidative stress, apoptosis, neuroinflammation), and their expression changes have been proposed as potential PD biomarkers [6]. Indeed, distinct cmiRNA signatures were shown to distinguish PD from atypical parkinsonian disorders and even to differentiate PD from multiple system atrophy in some studies. These findings underscore the biomarker potential of circulating miRNAs in PD [7,8].

Among the miRNAs implicated in PD, miR-146a-5p has drawn particular interest due to its role in regulating innate immunity and inflammation. MiR-146a is induced by NF-κB signaling and acts in a negative feedback loop to attenuate inflammatory pathways by targeting molecules in Toll-like receptor (TLR) and cytokine signaling. [9] In neural contexts, miR-146a-5p is considered a “neuroinflammatory regulator” that can be upregulated during chronic inflammatory stimulation of the brain [10]. Notably, in a rotenone-induced PD animal model, miR-146a was the most upregulated miRNA identified, triggered by NF-κB activation, and its upregulation led to reduced expression of the Parkin gene, linking inflammation to impaired mitophagy in dopaminergic neurons [11]. Such evidence suggests that miR-146a upregulation is part of the PD-related neuroinflammatory response, which might contribute to neurodegeneration if not properly regulated.

Clinical studies further support a role for miR-146a-5p in PD. MiR-146a-5p levels in blood have been associated with disease severity and duration. In one study, circulating miR-146a-5p was found to correlate positively with PD severity scores [12]. Another investigation reported that serum miR-146a-5p levels were significantly higher in female PD patients than in male patients at diagnosis, with a more than three-fold female-to-male difference. Intriguingly, that same study observed that miR-146a-5p expression had a significant positive correlation with disease duration in PD, particularly in women [13]. These findings hint that miR-146a-5p might increase as PD progresses, possibly more rapidly in females, although the cross-sectional design left open the question of individual longitudinal changes. More broadly, sex is an important biological factor in PD: men have a higher incidence of PD than women, and sex-linked factors (such as estrogen exposure or X-linked genetic modifiers) may influence disease course. Evidence of sex differences in miRNA expression in PD underscores the need to stratify analyses by sex when evaluating biomarkers. Based on the state-of-the-art data, we aimed to perform a novel exploration of the longitudinal behavior of circulating miRNAs in PD with attention to potential sex-specific patterns. We focused on a panel of miRNAs previously implicated in PD pathophysiology (including miR-146a-5p and others related to inflammation and neurodegeneration). In this study, early-stage PD patients were followed over time, and miRNA levels at baseline and follow-up were compared within each individual. By analyzing males and females separately, we sought to determine whether any miRNA changes were sex-dependent or common to both sexes. We report here that miR-146a-5p shows a striking and consistent upregulation over the disease course in both women and men with PD, suggesting that this miRNA could serve as a robust biomarker of PD progression and a window into disease mechanisms.

## 2. Results

### 2.1. Participant Characteristics

The study included 30 PD patients (22 men and 8 women) who completed both baseline and follow-up assessments. Demographic and clinical features of the study cohort are summarized in Table 1. All participants were levodopa-naïve at baseline and started levodopa treatment according to clinical judgment after baseline evaluation. At T2, subjects were on stable dopaminergic regimens for ≥6 months before sampling. Both Levodopa doses and Levodopa equivalent daily doses of other antiparkinsonian drugs were similar at T2 between men and women (*p* = 0.97, *p* = 0.45, respectively). To ensure that disease-stage variability did not influence the results, a sensitivity analysis excluding the single stage 3 subject was performed. Results remained unchanged, confirming the robustness of the findings. By the time of follow-up (approximately 2 years after baseline), all patients were still in early to mid-stages of PD; 5 patients (all originally drug-naïve) had initiated dopaminergic therapy during the interim, while the others remained on similar management as baseline. No participants were lost to follow-up.

### 2.2. Baseline miRNA Expression by Sex

We first compared serum miRNA levels between male and female PD patients at baseline (T0). Among the panel of miRNAs analyzed (miR-34a-5p, miR-146a-5p, miR-155-5p, miR-29a-3p, miR-106a-5p, etc.), none showed a statistically significant difference between sexes at T0. In particular, the baseline expression of miR-146a-5p was slightly higher in females than males on average (median 2^−ΔCt values were 0.021 vs. 0.018, respectively), but this difference did not reach significance (*p* = 0.20).

### 2.3. Longitudinal Change in miR-146a-5p

**Figure 1:**
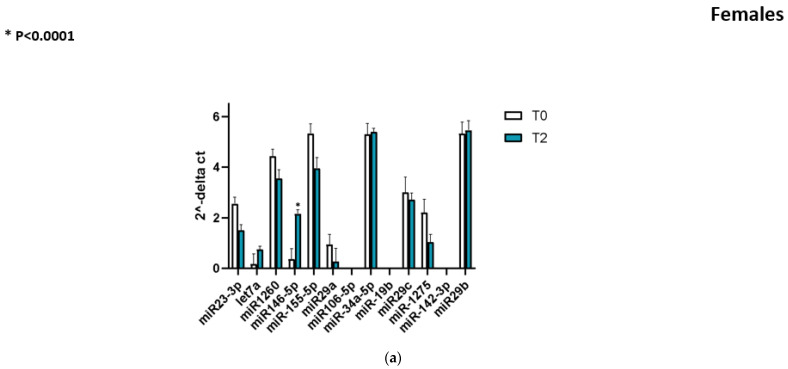

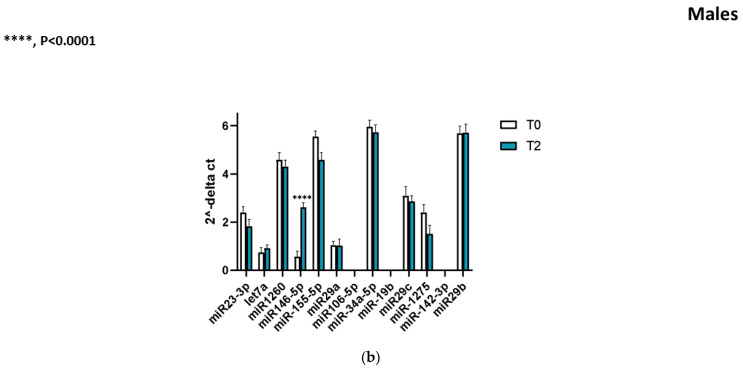
(**a**) Longitudinal changes in circulating miR-146a-5p in female MD patients. Serum miR-146a-5p levels (2^−ΔCt^, normalized to miR-93-5p) were measured at baseline (T0) and follow-up (T2). Each line connects paired values from the same patients. A significant upregulation of miR-146a-5p was observed at T2 compared with T0 (* *p* < 0.0001, paired test). (**b**) Longitudinal changes in circulating miR-146a-5p in male MD patients. Relative serum levels (levels (2^−ΔCt^, normalized to miR-93-5p) were significantly higher at follow-up (T2) compare with baseline (T0) (**** *p* < 0.0001, paired test).

A key finding of our study is that miR-146a-5p levels increased markedly from baseline to follow-up in both sexes. In female PD patients, serum miR-146a-5p expression at T2 was on average 5.96 times higher than at T0 (FC ≈ 5.96, *p* < 0.0001 for the paired comparison). Similarly, male PD patients showed a significant ~4.71-fold increase in miR-146a-5p from T0 to T2 (*p* < 0.0001). Figure 1a,b shown this pronounced upregulation, with each individual (male or female) showing higher 2^−ΔCt values at T2 than their own baseline. A comparison of ΔCq (miR-146a-5p) between levodopa monotherapy and combined regimens showed no significant differences (*p* > 0.5). There was no overlap in the distributions: virtually all patients had an increase, reflecting a consistent upward trajectory of miR-146a-5p expression over the course of disease. Importantly, the magnitude of the miR-146a-5p increase did not differ significantly between males and females (*p* = 0.38 for sex difference in Δ(2^−ΔCt)), indicating that both groups experienced a robust and comparable rise in this miRNA. Thus, the longitudinal pattern of upregulation was a shared phenomenon in both women and men with PD. No significant difference was found between the fold-change magnitudes of females and males. This consistent upward trend suggests a time-dependent elevation of miR-146a-5p during PD progression. Paired line plots connecting individual T0 and T2 ΔCt values (Figure 1a,b) illustrate a consistent decrease in ΔCt across both sexes (**** *p* < 0.0001), corresponding to a significant longitudinal upregulation of circulating miR-146a-5p.

In addition to hypothesis testing, we explored whether the degree of miR-146a-5p increase correlated with clinical progression. We found a moderate positive correlation between the fold change of miR-146a-5p and the increase in MDS-UPDRS III motor score from T0 to T2 (Spearman ρ ≈ 0.40, *p* = 0.03), although this did not survive correction for multiple comparisons and should be interpreted cautiously. No clear correlation was observed between miR-146a-5p changes and changes in non-motor symptom scores. Nonetheless, the overall picture is that miR-146a-5p expression rises substantially as PD advances, in parallel with worsening motor symptoms in some patients.

### 2.4. Other miRNAs

We evaluated longitudinal changes for other candidate miRNAs in our panel. In contrast to miR-146a-5p, none of the other tested miRNAs showed a statistically significant change from baseline to follow-up in either sex. For example, miR-34a-5p (which was higher in males at baseline in a prior study) did not exhibit any consistent directional change over time in our cohort (female FC 1.1, male FC 1.0, *p* > 0.5). MiR-155-5p, another key regulator of immune pathways, showed a trend toward increase at T2, but this did not reach significance (male FC ~1.3, *p* = 0.15; female FC ~1.5, *p* = 0.10). MiR-29a-3p and miR-106a-5p were essentially unchanged longitudinally. Thus, the miR-146a-5p result stood out as the clearest and most robust longitudinal alteration among the miRNAs examined. We also confirm that at baseline, none of those miRNAs differed between men and women (as noted above), in line with our primary sex-stratified analysis.

## 3. Discussion

In this study, we provide evidence that circulating miR-146a-5p is markedly upregulated over time in PD patients, in both women and men. Using a longitudinal design with paired baseline and follow-up samples, we observed a nearly six-fold increase in miR-146a-5p expression in female PD patients and a nearly five-fold increase in male patients after approximately two years of disease progression. To our knowledge, this is the first demonstration of a longitudinal miRNA change within the same PD individuals, indicating that miR-146a-5p may dynamically reflect the course of the disease. This finding is particularly noteworthy given that baseline sex differences in miR-146a-5p (and other miRNAs in our panel) were not significant in our cohort—yet the temporal trajectory of miR-146a-5p was strikingly consistent across sexes. Our results highlight miR-146a-5p as a candidate biomarker for PD progression and shed light on the biological processes that may intensify as the disease advances. This biomarker may present an important tool to assess efficacy of new therapeutics, which will require further studies.

### 3.1. Comparison with Previous Studies

The pronounced rise in miR-146a-5p observed in our study aligns with prior cross-sectional evidence linking this miRNA to PD duration and severity. Vallelunga et al. (2023) reported that, at diagnosis, female PD patients had higher serum miR-146a-5p than males, and they observed a positive correlation between miR-146a-5p levels and disease duration [13]. Our longitudinal data confirm that miR-146a-5p increases as disease duration lengthens, supporting the notion that miR-146a-5p is a progression-related biomarker. Interestingly, in our smaller sample, we did not detect the baseline sex disparity seen in the larger 2023 cohort. However, at follow-up, both sexes showed elevated miR-146a-5p, suggesting that even if women start at a somewhat higher level (as reported in the literature), men may “catch up” as the disease progresses. The net effect is that miR-146a-5p appears to rise in everyone with PD over time, which is consistent with an intrinsic disease-driven process rather than a sex-specific effect.

The behavior of miR-146a-5p in PD has been somewhat heterogeneous in different studies, especially in cross-sectional case–control comparisons. For example, one study found miR-146a-5p to be downregulated in the blood of PD patients relative to healthy controls, which at first glance seems at odds with our findings of an increase over time [5]. However, that earlier study largely examined treated PD patients at a single time point. It is possible that in early PD, miR-146a-5p is relatively low (perhaps indicating an inadequate anti-inflammatory response in the initial disease stages), and as PD progresses, miR-146a-5p is upregulated as part of a compensatory or disease-exacerbating response. This dynamic could reconcile discrepancies: early PD might feature a deficit in miR-146a-mediated regulation, whereas later PD sees an overactivation. Longitudinal tracking, as done here, is crucial for capturing such temporal patterns that cross-sectional studies cannot resolve.

### 3.2. Biological Significance of miR-146a-5p Upregulation

MiR-146a is well known as a key regulator of innate immunity and inflammation. It is transcriptionally upregulated by NF-κB in response to pro-inflammatory signals and in turn targets multiple components of the Toll-like receptor (TLR) and cytokine signaling cascade (including IRAK1, TRAF6) to dampen excessive inflammation. In the context of neurodegeneration, miR-146a is thought to be induced in glial cells (microglia and perhaps astrocytes) during chronic inflammation, as a mechanism to limit inflammatory injury [9,10]. Our finding of rising miR-146a-5p levels in PD over time suggests that chronic neuroinflammation is mounting as the disease progresses. Indeed, PD is characterized by activated microglia and sustained inflammation in the brain; miR-146a may be upregulated in peripheral blood immune cells or released into circulation as part of this process. Notably, Jauhari et al. demonstrated in a PD toxin model that NF-κB-driven miR-146a induction can negatively regulate the Parkin gene, which is crucial for mitophagy and neuronal health [11]. This raises a provocative possibility: as PD progresses, increasing miR-146a-5p might reflect an ongoing inflammatory response, which initially aims to counteract inflammation but might have deleterious side effects such as impairing mitophagy via Parkin downregulation. Thus, chronic elevation of miR-146a-5p could potentially contribute to worsening neurodegeneration despite its anti-inflammatory intent, creating a vicious cycle.

Furthermore, miR-146a-5p has been linked to other neurodegenerative pathways. For instance, it can influence signaling of TNF-α and other cytokines, and it has been found upregulated in models of Alzheimer’s disease and other conditions with innate immune activation [14,15]. In PD patients, higher miR-146a-5p has been associated with more severe disease, reinforcing the idea that it tracks with an advancing pathogenic process [13]. Our observation that the increase in miR-146a-5p correlated with motor progression (change in UPDRS scores) in our cohort, albeit modestly, supports a link between this miRNA and clinical worsening. However, larger studies would be needed to confirm the strength of this correlation.

It is also instructive to consider miR-146a-5p alongside other inflammation-related miRNAs in PD. We examined miR-155-5p, another pro-inflammatory microRNA that is often co-regulated with miR-146a in immune cells. MiR-155 has been shown to drive inflammatory responses and is elevated in PD blood and brains. Intriguingly, one report found that miR-155 levels in PD patients were affected by levodopa therapy, with high-dose treatment associated with lower miR-155 [3,5]. In our study, miR-155-5p did not significantly increase over time (only a trend), which might suggest that any longitudinal change could be blunted by treatments or that miR-155 behaves differently than miR-146a in the progression context. MiR-146a-5p, being more of a negative feedback regulator, might be uniquely sensitive as a readout of cumulative inflammatory activation. Our results hint that miR-146a-5p could be a more robust indicator of the chronic state of inflammation in PD, whereas miR-155 might be more acutely modulated by ongoing stimuli or therapies. This could explain why miR-146a-5p showed a clearer trajectory in our longitudinal analysis.

### 3.3. Implications for Biomarkers and Therapy

The strong and consistent upregulation of miR-146a-5p in PD patients over time has potential implications for both biomarker development and therapeutic strategies. As a biomarker, miR-146a-5p could be explored as a non-invasive indicator of disease progression. Currently, clinicians lack a blood test to gauge how quickly a patient’s PD is advancing. If validated in larger cohorts, monitoring miR-146a-5p periodically could complement clinical assessments: a significant rise might signal accelerating progression or heightened neuroinflammatory activity. Moreover, because miR-146a-5p is measured in serum, this could be incorporated into studies of disease-modifying therapies to see if treatments can alter the trajectory of this biomarker. For example, an anti-inflammatory or neuroprotective therapy that dampens the pathological processes in PD might also prevent the usual rise of miR-146a-5p—thus miR-146a-5p could serve as a pharmacodynamic marker.

From a therapeutic angle, miR-146a itself might be a target of interest. Since miR-146a-5p serves to restrain inflammation, one might argue that its upregulation is a beneficial response that should perhaps be augmented. On the other hand, if excessive miR-146a activity impairs important targets like Parkin, then inhibiting miR-146a could be neuroprotective. The situation is complex, and likely time-dependent. Early in PD, relatively low miR-146a-5p might contribute to unchecked inflammation; later on, very high miR-146a-5p might indicate a system struggling to counteract inflammation but inadvertently affecting other pathways. Any therapeutic modulation of this miRNA would need to carefully tune the balance. Nonetheless, our findings add to a growing recognition that miRNAs like miR-146a and miR-155 are central players in PD-related inflammation and could be harnessed in the future. For instance, miR-155 is already considered a promising target for anti-inflammatory therapy in PD. MiR-146a, given its broad regulatory reach, might also be a candidate for therapeutic upregulation (using miRNA mimics) or downregulation (using anti-miRs), depending on further preclinical studies.

### 3.4. Limitations

We acknowledge several limitations in our study. The sample size, particularly the number of female patients, was relatively small (only eight women), which limits the power to detect subtle sex differences and may limit generalizability. Our cohort comprised early-stage PD patients, mostly drug-naïve at baseline, which is a strength for observing disease-intrinsic changes but means the results may differ in later-stage or treated populations. While the follow-up of ~2 years was sufficient to capture a clear change in miR-146a-5p, additional time points (including an intermediate time, e.g., 1 year) could have helped map the trajectory more finely; it is unknown whether miR-146a-5p increases linearly or perhaps accelerates at a certain disease stage. We focused on a candidate-based panel of miRNAs, so it’s possible we missed other relevant miRNAs that might change over time; unbiased profiling (e.g., small RNA sequencing) was beyond the scope of this study but could reveal a broader landscape of longitudinal miRNA alterations. Another consideration is that some patients started dopaminergic therapy during the follow-up, which could influence miRNA levels (as suggested for miR-155 in prior work). However, the consistent increase in miR-146a-5p across all patients argues that the effect is disease-driven rather than medication-driven. Still, future studies should control for medication status more rigorously. Finally, we did not have parallel cerebrospinal fluid or brain tissue samples to directly link peripheral miRNA changes with central changes. It remains an open question whether increased serum miR-146a-5p reflects increased brain miR-146a expression or is a marker of peripheral immune cell activity; likely it is a combination of both, given that miR-146a is induced in monocytes and microglia alike.

## 4. Materials and Methods

### 4.1. Study Participants

Thirty patients with idiopathic Parkinson’s disease were enrolled in this longitudinal study. All patients met the MDS clinical diagnostic criteria for PD. Key inclusion criteria included early to mid-stage disease (Hoehn & Yahr stage 1–3 at baseline) and no history of other neurological disorders. At baseline (T0), there were 22 males and 8 females, with a mean age of approximately 64 years. The median disease duration at T0 was 14.5 months, reflecting an early disease cohort. Patients were evaluated again at a follow-up time point (T2); the follow-up interval was approximately 24 months after baseline for all participants. During the study period, patients received standard clinical care; any changes in treatment were recorded, although the cohort initially included levodopa-naïve patients. All patients provided written informed consent prior to enrollment. The study protocol (AIFA code: 2016-02364714) was reviewed and approved by the Campania Sud Ethics Committee, in compliance with the principles of the Declaration of Helsinki.

### 4.2. Sample Collection and Processin

Fasting blood samples were collected from each participant at T0 and T2 via venipuncture into serum separator tubes. Samples were processed within 2 h of collection to isolate serum. Blood tubes were allowed to clot at room temperature for ~30 min, then centrifuged at 3000 rpm for 15 min at 4 °C. The resulting serum was aliquoted and stored at −80 °C until analysis. To ensure sample quality, we assessed hemolysis in each serum sample by measuring the ratio of miR-451a to miR-23a-3p as described by Shah et al. [16] Samples with evidence of more than mild hemolysis (miR-451a/23a ratio > 5) were excluded from analysis, as hemolysis can release confounding erythrocyte miRNAs.

### 4.3. miRNA Extraction and Quantification

Total RNA enriched for small RNAs was extracted from 200 µL of serum using a silica membrane column kit optimized for miRNA (for example, Qiagen miRNeasy Serum/Plasma Kit (Qiagen, Hilden, Germany) or similar), following the manufacturer’s instructions. A non-human spike-in control miRNA was added to each sample before extraction to monitor technical yield. RNA was eluted in a small volume of RNase-free water and stored at −80 °C. For cDNA synthesis, we employed a TaqMan Advanced miRNA cDNA Synthesis Kit (Thermo Fisher, Waltham, MA, USA) or a universal cDNA synthesis kit for miRNAs, per protocol. Quantitative real-time PCR (qPCR) was then performed using locked nucleic acid (LNA)–enhanced miRNA-specific assays (Exiqon/Qiagen) for each target miRNA. Each sample was run in duplicate PCR reactions; the average cycle threshold (Ct) value of the duplicates was used for analysis to ensure accuracy. The panel of miRNAs assayed included miR-146a-5p and several other candidates (e.g., miR-34a-5p, miR-155-5p, miR-29a-3p, miR-106a-5p) based on prior associations with PD.

Normalization was performed using miR-93-5p, which displayed high expression stability according to geNorm- and NormFinder-based analyses. To confirm robustness, data were re-analyzed using the geometric mean of miR-93-5p and the most stable reference candidate identified by the stability ranking, yielding highly concordant results (Pearson *r* ≈ 0.99; Supplementary Figures S1 and S2). The relative expression of each target miRNA was calculated using the 2^−ΔCt method, where ΔCt = Ct_(target miRNA)—Ct_(miR-93-5p). A lower ΔCt (or higher 2^−ΔCt value) corresponds to higher abundance of the target miRNA. For longitudinal comparisons, fold change (FC) between T2 and T0 was computed as 2^−ΔCt(T2)/2^−ΔCt(T0) for each individual, and group-level geometric means were used to summarize fold changes [17].

### 4.4. Statistical Analysis

All statistical analyses were performed with GraphPad Prism 10 (GraphPad Software, San Diego, CA, USA). We first examined the distribution of miRNA expression values (2^−ΔCt) for normality using the D’Agostino-Pearson test. As miRNA data are often not normally distributed, non-parametric tests were emphasized when appropriate. To compare baseline characteristics between male and female patients (such as age or clinical scores), we used t-tests or Mann–Whitney U tests as appropriate. For baseline miRNA expression differences between sexes, we used the Mann–Whitney U test (given the relatively small sample size and non-normal distribution of some ΔCt values). Longitudinal changes in miRNA levels within the same individuals (T2 vs. T0) were assessed using paired statistical tests (the Wilcoxon signed-rank test for most miRNAs, which did not meet normality assumptions). In the case of miR-146a-5p, which showed a very pronounced change, we confirmed significance with a paired t-test as well, given the magnitude of effect. Significance was defined at the 2-tailed α = 0.05 level. For the primary longitudinal comparisons, we report exact *p* values and fold changes. We did not apply a formal correction for multiple comparisons to the miRNA panel results, given the exploratory nature of this analysis and the limited number of miRNAs tested, but we highlight findings that meet stringent significance thresholds (*p* < 0.0001). In addition, we evaluated correlations between baseline miRNA levels and clinical variables (disease duration, UPDRS-III motor score) using Spearman’s correlation coefficient [17]. All participants were levodopa-naïve PD patients at baseline. Longitudinal data were visualized using paired line plots (spaghetti plots) to preserve within-subject pairing and depict inter-individual variability, as recommended for biomarker studies [18,19].

## 5. Conclusions

Our findings demonstrate a robust longitudinal upregulation of miR-146a-5p in the blood of PD patients, in both women and men, suggesting that this miRNA is intimately linked with the progressive pathophysiology of PD. This sex-independent rise of miR-146a-5p over time provides a new perspective on previously observed sex differences, indicating that dynamic changes may converge even if baseline levels diverge. MiR-146a-5p stands out as a potential biomarker of disease progression, warranting further study in larger cohorts and in relation to clinical outcomes. Moreover, given miR-146a’s role in modulating inflammatory networks, these results reinforce the concept that neuroinflammation is a driving component of PD progression. In conclusion, our longitudinal data indicate that circulating miR-146a-5p undergoes a significant upregulation over the two-year follow-up in early PD patients, independent of age or therapy.

This observation is biologically plausible given the established role of miR-146a-5p as a master regulator of microglia-mediated neuroinflammatory signaling and immune homeostasis in the central nervous system [20]. Altered circulating levels of this miRNA have also been reported in PD cohorts [21], and recent reviews have emphasized its diagnostic and prognostic relevance in neurodegenerative disorders [22]. Moreover, evidence that miR-146a-5p is stably detectable in plasma across multiple time points [1] further supports its technical suitability as a dynamic, minimally invasive biomarker for disease monitoring. Altogether, these findings strengthen the interpretation that miR-146a-5p reflects ongoing neuroinflammatory activity associated with PD progression.

Therapies targeting inflammatory pathways—possibly including miRNA-based interventions—could hold promise for slowing the course of PD. Our work adds a piece to the puzzle of PD biomarkers and underscores the value of longitudinal studies in uncovering the temporal dimensions of molecular changes in Parkinson’s disease. Future research should expand on these findings, exploring how miR-146a-5p and other key miRNAs might be integrated into multi-marker panels to improve monitoring of PD and to open new avenues for therapeutic modulation of disease pathways.

## Figures and Tables

**Table 1:** Clinical and demographic characteristics of Parkinson’s disease patients at baseline (T0).

	PD Patients (n = 30)
Sex (M/F)	22/8
Age (years) (Mean ± SD)	65.9 ± 10.3
BMI (kg/m^2^) (Mean ± SD)	25.2 ± 4.2
Disease duration (months) (Mean [Range])	22 (2–120)
MDS-UPDRS III total score (Mean ± SD)	28 ± 12
Hoehn & Yahr stage	2 (1–3)

Values are presented as mean ± SD or median (range) as appropriate. Abbreviations: BMI = Body Mass Index; MDS-UPDRS = Movement Disorder Society Unified Parkinson’s Disease Rating Scale.

## Data Availability

The data presented in this study are available on request from the corresponding authors due to ethical restrictions.

## Supplementary Materials

The following supporting information can be downloaded at: https://www.mdpi.com/article/10.3390/ijms262110315/s1.
