# Supplementary figures and images for: Sex-Independent Upregulation of miR-146a-5p in Parkinson’s Disease Patients: A Longitudinal Study
Source: Int J Mol Sci. 2025 Oct 23;26(21):10315. doi: 10.3390/ijms262110315 (PMC12610492; doi:10.3390/ijms262110315)

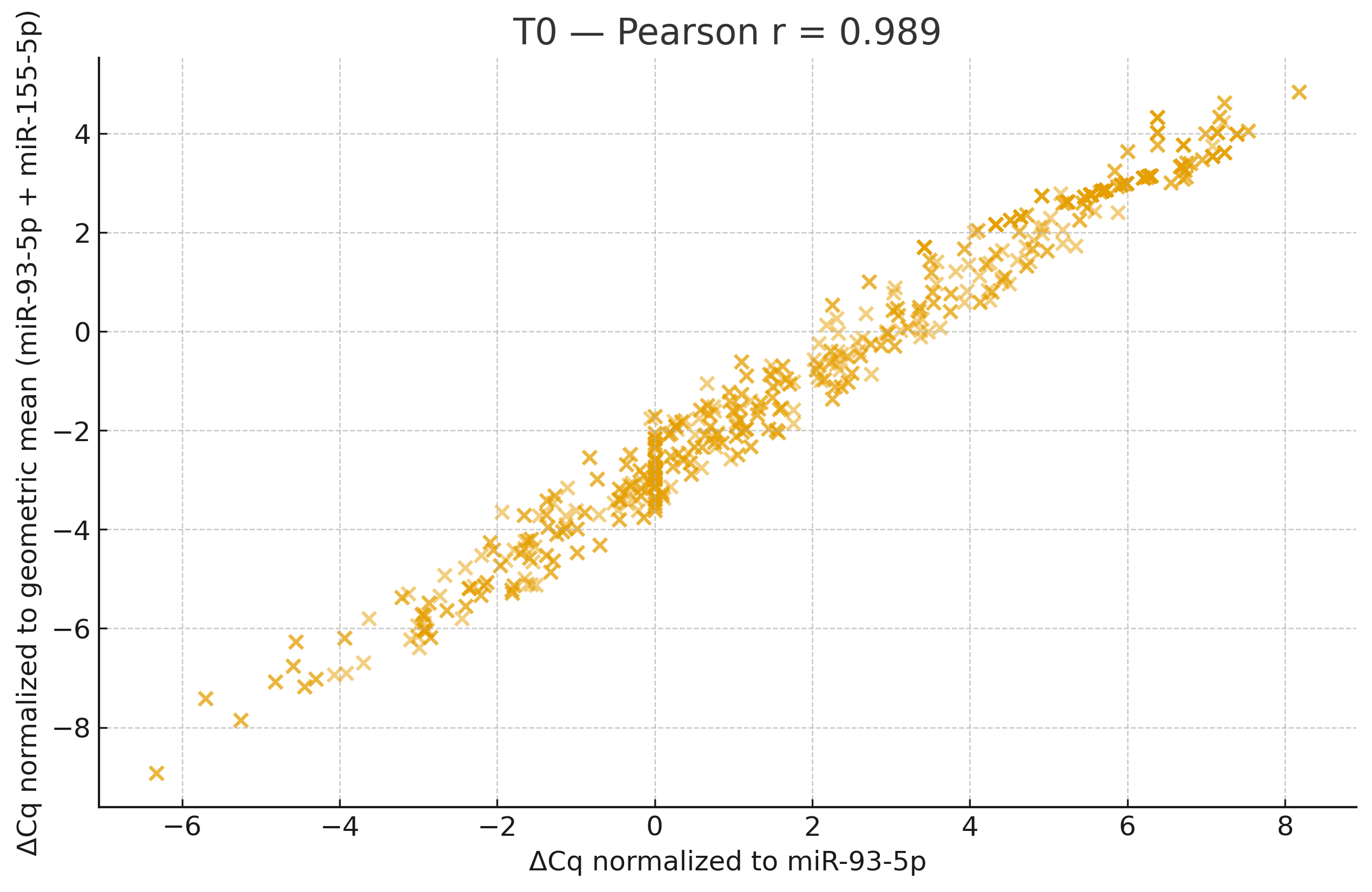

Supplement: Supplementary file 1 [file ijms-26-10315-s001.zip › Figure S1_robustness_scatter_T0.png]

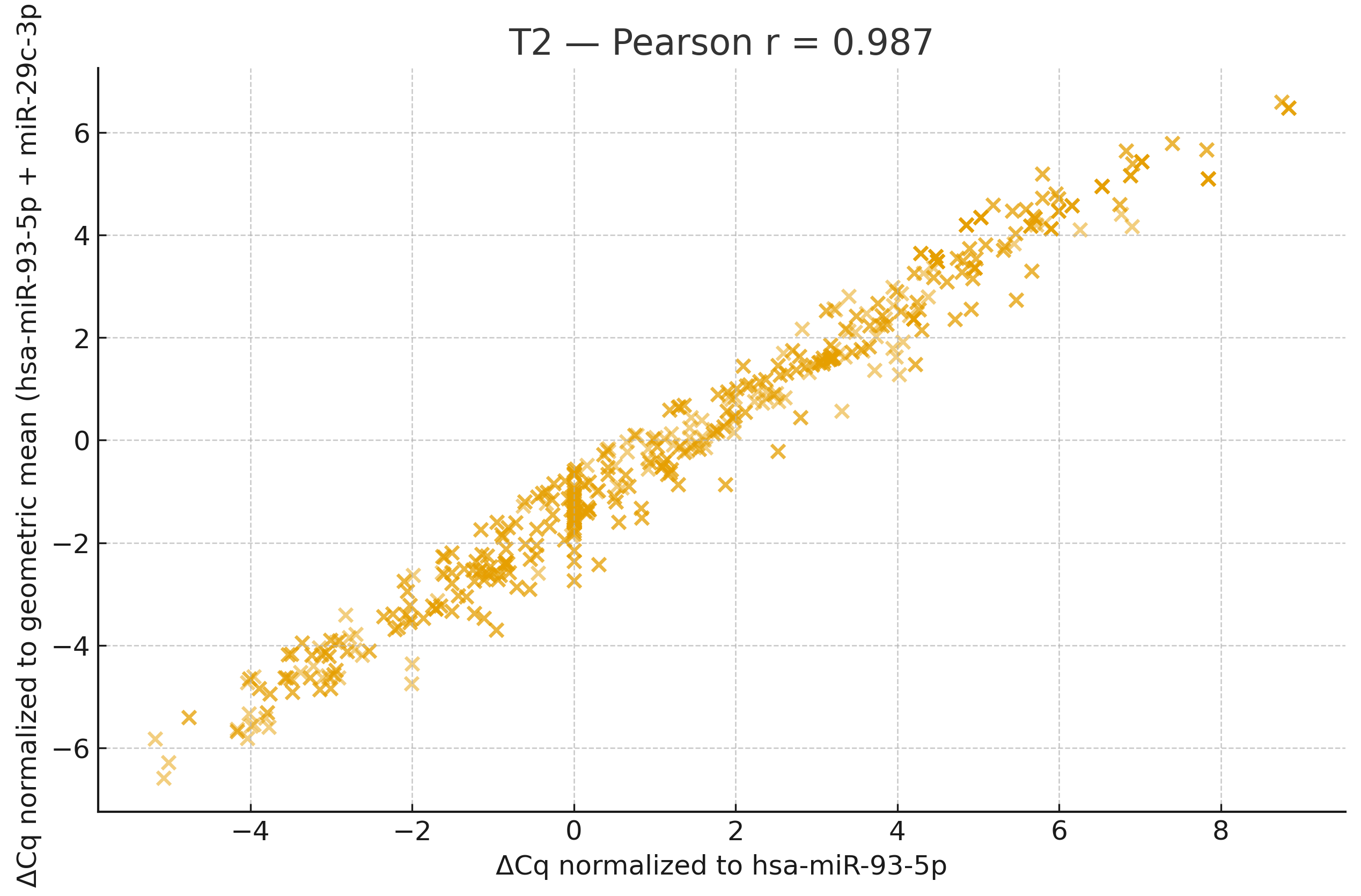

Supplement: Supplementary file 1 [file ijms-26-10315-s001.zip › Figure S2_robustness_scatter_T2.png]
